# Single dose of morphine produced a prolonged effect on dopamine neuron activities

**DOI:** 10.1186/1744-8069-4-57

**Published:** 2008-11-17

**Authors:** Die Zhang, Hai Zhang, Guo-zhang Jin, Kehong Zhang, Xuechu Zhen

**Affiliations:** 1 State Key Laboratory of Drug Research, Department of Neuropharmacology, Shanghai Institute of Material Medica, Chinese Academy of Sciences, Shanghai, PR China

## Abstract

**Background:**

Clinical observation and experimental studies have indicated that a single exposure to morphine could induce tolerance and dependence. It has become a concern in clinical antinociceptive practice. However, the underling mechanism remains unknown. This study was designed to explore the changes of dopamine (DA) neuron activities in the ventral tegmental area (VTA) by employing a spectral analysis followed single morphine treatment.

**Results:**

Acute morphine treatment significantly increased not only the firing rate and firing population but also the power of slow oscillation of DA neurons in naïve rats. These changes lasted at least for 3 days following the morphine treatment. During this period of time, responses of the DA neurons to subsequent morphine challenge were diminished. We further demonstrated a transient desensitization of opiate receptors as monitored by GTPγS binding to G-proteins. The present study provided first direct evidence for the temporal changes in the VTA DA neuron activities and opiate receptors desensitization.

**Conclusion:**

Prolonged VTA DA neuron activation and opiate receptors desensitization followed single morphine exposure may underlie the development of dependence and tolerance that may associate with the acute analgesic tolerance and acute addiction of morphine.

## Introduction

Dopamine (DA) neurons in the ventral tegmental area (VTA) project primarily to the nucleus accumbens and the prefrontal cortex, forming the mesocorticolimbic system [1-3]. Like other drugs of abuse, morphine influence DA neurotransmission directly or indirectly[4,5]. Positive reinforcement of drugs of abuse is believed to be mediated through the activation of the mesocorticolimbic DA system[6]. Repeated exposure to morphine induce substantial adaptive changes in cellular and synaptic functions in the mesocorticolimbic DA system [7,8]. These adaptations are believed to play a critical role in the development of tolerance and dependence [9,10].

Clinical and experimental evidences have revealed that a single exposure to morphine could induce rapid tolerance and dependence that may associate with acute addiction and analgesic tolerance [11-14]. Increased firing activity of VTA DA neurons in response to acute morphine administration has been reported [15-17]. However, the long-lasting effect and the time course (onset, duration, and dissipation) on the activity of VTA DA neurons and how this change may contribute to the single morphine exposure-induced dependence or tolerance that may associate with decreased analgesic potency for morphine remains inadequately defined.

In the present study, we examined the firing activities of the VTA DA neurons at various time points after a single morphine injection. In addition to the traditional electrophysiological parameters such as firing rate and bursting, we also included slow oscillation (SO), SO represents a novel and major firing pattern of DA neurons in the VTA [18]. The SO DA neurons is characterized by a low frequency oscillation at 0.5–1.5 Hz [18,19]. Previous works in this laboratory and others have demonstrated that nearly 50 percentage of VTA DA neurons exhibits SO in basal condition and psychostimulants were found to alter SO [20,21]. Very recently, we demonstrated that SO in the VTA DA neurons is largely dependent on the input from the prefrontal cortex (PFC)[22], further suggesting the importance of SO in VTA DA neuron functional regulation.

The results from the current study indicated that the firing rate, bursting, SO, and the firing population of the VTA DA neurons in rats remain increased for at least 3 days after a single morphine exposure. Within this limited window of time, the VTA DA neurons failed to respond to additional morphine challenge. Indicating a transient morphine tolerance in VTA DA neuron activity in rats was developed with a single dose of morphine treatment. We further demonstrated that the acute morphine tolerance was associated with impairment of opiate receptor-G protein coupling, indicating that down regulation of G-protein activation may contribute to acute morphine tolerance.

## Methods

### Materials

Morphine hydrochloride was purchased from Qinghai Pharmaceutical Factory (Qinghai, China) and dissolved in saline. [^35^S] GTPγS (1050 Ci/mmol) was purchased from Amersham Biosciences (Piscataway, USA). Other chemicals were obtained from standard suppliers.

### Animal Preparations

All procedures were in compliance with the Guidelines for the Care and Use of Laboratory Animals (National Research Council, People's Republic of China, 1996). Male Sprague-Dawley (SD) rats, with an initial body weight of 270 to 300 g, were maintained on a 12 h light (8:00 am-8:00 pm)/12 h dark schedule at a temperature of 22 ± 2°C and 65% humidity. Access to standard food and water was unlimited. Rats were acclimated to the animal facility for at least 7 days before experiments. All experiments were conducted between 10:00 am and 4:00 pm. For the initial exposure, morphine was given subcutaneously (s.c.) at a dose of 20 mg/kg in a volume of 2 ml/kg. For subsequent challenge, morphine was given intravenously (i.v.) at a dose of 5 mg/kg in a volume of 1 ml/kg[23,24].

### Single Unit Recording

Rats were anesthetized with choral hydrate (400 mg/kg, i.p.) and mounted in a stereotaxic apparatus (Narishige, Japan). Body temperature was maintained at 36–37°C with a homeothermic blanket system (Harvard, USA). Glass microelectrodes were filled with 2 M NaCl and 0.2% pontamine Sky Blue as a tracer. Resistance ranged from 6 to 8 MΩ. Electrode was placed into the VTA through a small burr hole in the skull (3.0 mm anterior to the lambda and 0.5–0.9 mm lateral to the midline) by an electro-microdriver. DA neurons, usually at 6.5–8.5 mm below the cortical surface, were identified according to the published criteria[19,21,25]: a biphasic (positive-negative) or triphasic (positive-negative-positive) spike waveform with a duration of greater than 3.0 ms, a broad initial positive phase (>1 ms, measured from the start of action potential to the negative trough), an initial segment-somatodendritic break in the initial positive phase, and a slow firing rate (<10 spikes/s). In addition, DA neurons can also be distinguished from non-DA neurons by the characteristic low-pitched sound that they produce on an audio monitor. Data were collected and analyzed using an acquisition system by the Fudan University (Shanghai, China) and stored in a computer for off-line analysis. After recording, rats were killed and brains were kept for histological exam to verify the location.

### Population Recording (cells-per-track)

The number of spontaneously firing DA neurons within the VTA was determined by lowering the electrode through a area of tissue (400 × 600 μm). Twelve passes separated by 200 μm were made in each rat. The sequence of the passes and anatomical boundary of recorded area were identical among experimental subjects[19,21]. Only neurons with electrophysiological characteristics matching those previously established for midbrain DA neurons were included in the count. Each neuron was recorded and analyzed online for 2 to 5 minutes. The average number of spontaneously firing DA neurons encountered per electrode track (cells-per-track) was taken as an index for population activity of the VTA DA neurons.

### Membrane preparation

Rats were sacrificed by decapitation. The brain was removed and immediately homogenized in 20 volumes of ice-cold 300 mM sucrose solution using a teflon/glass homogenizer. The homogenate was centrifuged at 1,000 g for 10 min at 4°C. The supernatant was then centrifuged at 36,000 g for 30 min. The pellet was washed in 50 mM Tris (pH 7.5) and centrifuged again at 36,000 g for 30 min. The final pellet was suspended in 50 mM Tris (pH 7.5). Protein concentration was determined using a BCA method.

### GTPγS-binding assay

GTPγS-binding was performed in a reaction buffer containing: 50 mM Tris (pH7.5), 5 mM MgCl_2_, 1 mM EDTA, 100 mM NaCl, and 1 mM DTT, 0.1 nM [^35^S]GTPγS, and 30 μM GDP. Basal binding was determined in the absence of morphine. 10 μM of morphine was used to determine the morphine-stimulated GTPγS-binding. The assay was started by adding membrane preparation containing 15 μg protein to 100 μl reaction buffer. After 60 min of incubation at 30°C, the reaction was stopped with 3 ml ice-cold buffer. Samples were filtered rapidly through GF/C filters. Radioactivity in the filters was measured by liquid scintillation spectrometry. Nonspecific binding was determined using 10 μM unlabeled GTPγS.

### Data Analyses

Data was analyzed off-line with a software package from BSPS Mathlib (Fudan University; Shanghai, China). The firing rate, bursting, and coefficient of variation(CV) were analyzed every 10 sec as described previously[19,22]. SO was analyzed using a program developed by this laboratory[21]. Data were analyzed by using either independent *t*-test or ANOVA. Spectral data were log-transformed before statistical analysis. All measures were based on a 2-min recording for each subject. All numerical results are expressed as mean ± SEM.

## Results

### Acute morphine alters firing rate and firing pattern of the VTA DA neurons in naïve rats

**Figure 1:**
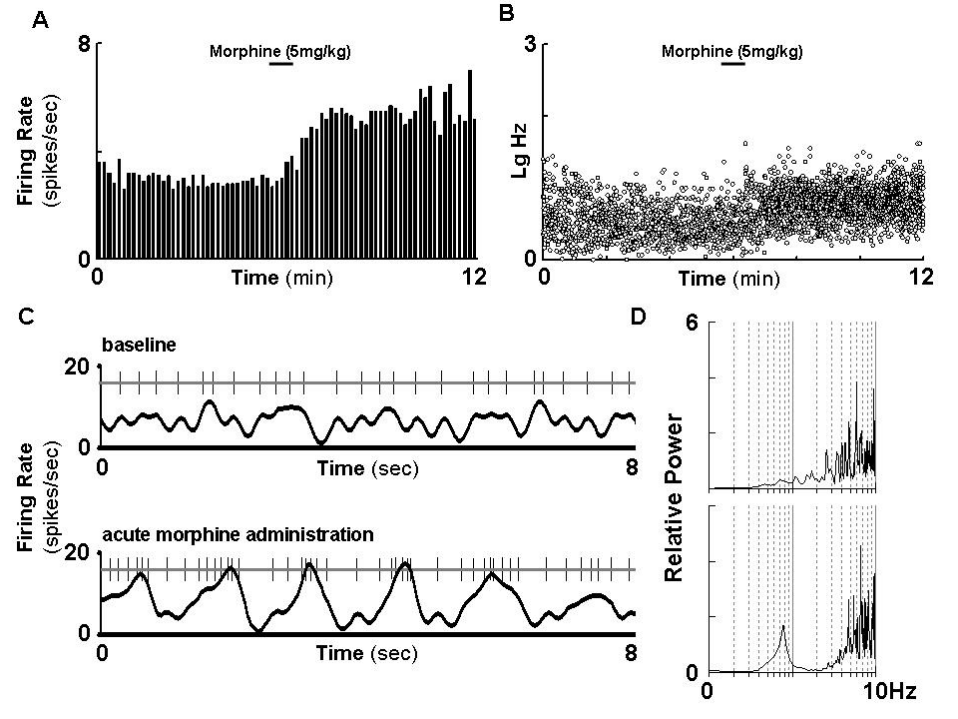
**Morphine alters the firing rate and pattern of the VTA DA neurons in naïve rats**. The firing activities were recorded before and after morphine injection (5 mg/kg, i.v.). A: firing rate of a representative DA neuron. B: distribution of firing rate in the same neuron. C: representative segment of firing spike train recorded from a DA neuron before and after morphine treatment. D: spectral chart before (upper panel) and after (lower panel) morphine treatment. SO: slow oscillation.

Intravenous injection of morphine at a dose of 5 mg/kg significantly increased the firing rate of the VTA DA neurons (+21.1 ± 8.6%, n = 8, paired *t*-test, p < 0.01; Figure. 1). Bursting (percentage of spikes firing in burst) and the coefficient of variation (CV) of interspike intervals (ISIs) were not affected. Moreover, the spectral analysis showed that acute morphine injection greatly enhanced VTA DA neurons slow oscillation (SO) power (+115.7 ± 58.8%, Paired t test, p < 0.05). This indicates that acute morphine not only enhanced the firing rate of VTA DA neurons, but also altered the firing patterns.

### Single morphine exposure induces prolonged changes of VTA DA neuron activity

**Figure 2:**
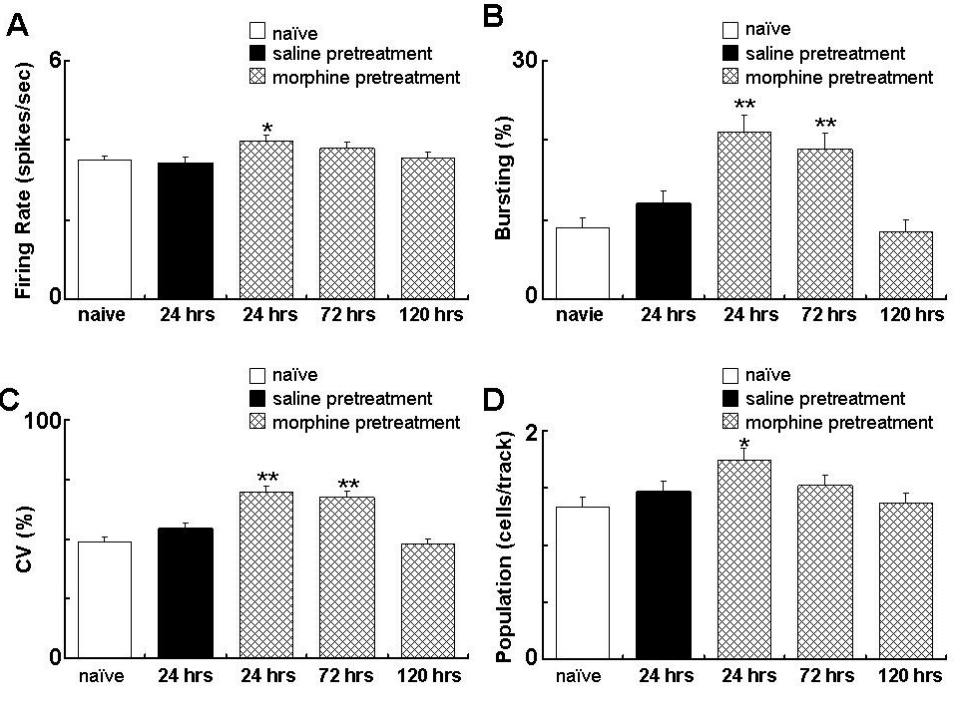
**Single morphine exposure produces a prolonged change on the firing activities of the VTA DA neurons**. Rats received morphine (20 mg/kg, s.c.) or saline. The firing activities were recorded at 24, 72 and 120 hours after morphine treatment. Naïve rats were used to indicate the basal activity. A: firing rate. B: bursting. C: CV of ISIs. D: cells/track. *P < 0.05, **P < 0.01, compared to saline control. Independent group *t*-test.

**Figure 3:**
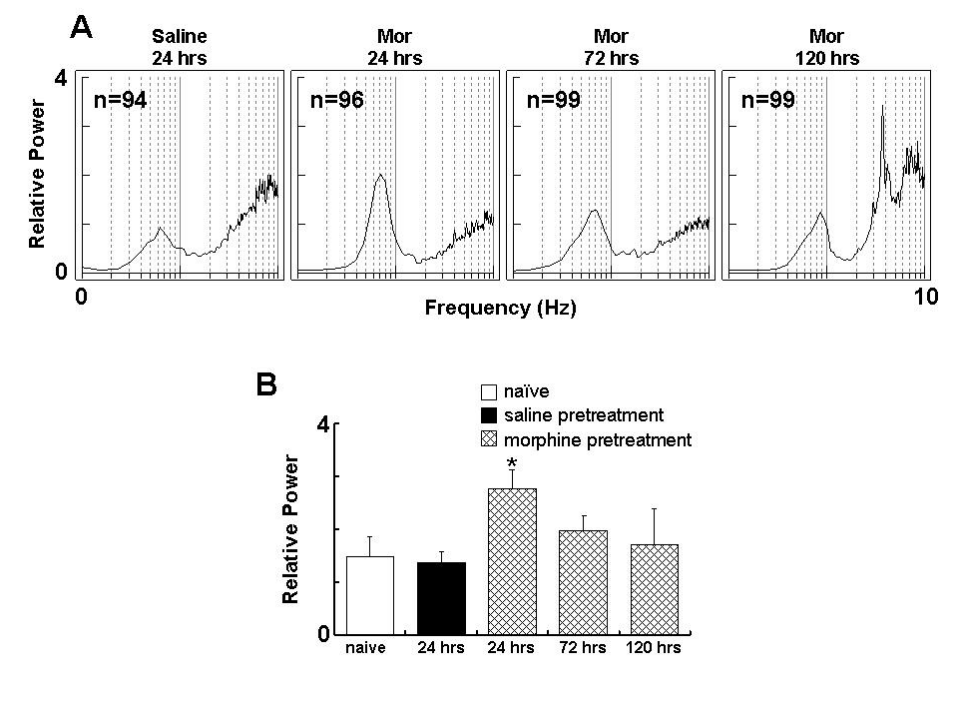
**Single morphine exposure alters firing pattern of VTA DA neurons**. Rats received morphine (20 mg/kg, s.c.) or saline. A: spectral charts at 24, 72, 120 hours after the treatment. B: group summary. *P < 0.05, compared to saline control. Mor: morphine.

As depicted in Figure. 2 &3, at 24 hours after morphine exposure (20 mg/kg, s.c.), firing rate of the VTA DA neurons was significantly higher than that in the control rats receiving saline (3.99 ± 0.14 spikes/sec, *t*-test, p < 0.05, compared to saline control). Bursting (20.96 ± 2.12%, p < 0.001), CV of ISIs (69.66 ± 2.40%, p < 0.001) and the number of firing VTA DA cells/track (1.74 ± 0.10 cells/track, p < 0.05). SO power (1.39 ± 0.17, p < 0.05, compared to control) were also significantly higher than the corresponding measures in the control rats receiving saline.

At 72 hours after the exposure, the firing rate, SO power and the number of firing DA neurons were no longer significantly different from the saline control However, the bursting level and CV of ISIs were still significantly higher than that in the saline control (bursting: 18.87 ± 2.05%, p < 0.001; CV: 67.63 ± 2.34%, p < 0.001). At 120 hours, all recorded parameters of the VTA DA neurons returned to a level comparable to that in the saline control group and are significantly lower than that of corresponding measurement obtained at 24 hours after morphine treatment. This result indicates that single dose of morphine induced a prolonged response in VTA DA neuron activity which lasted at least 72 hours.

### VTA DA neurons fails to response to subsequent challenge after the initial exposure to morphine

**Figure 4:**
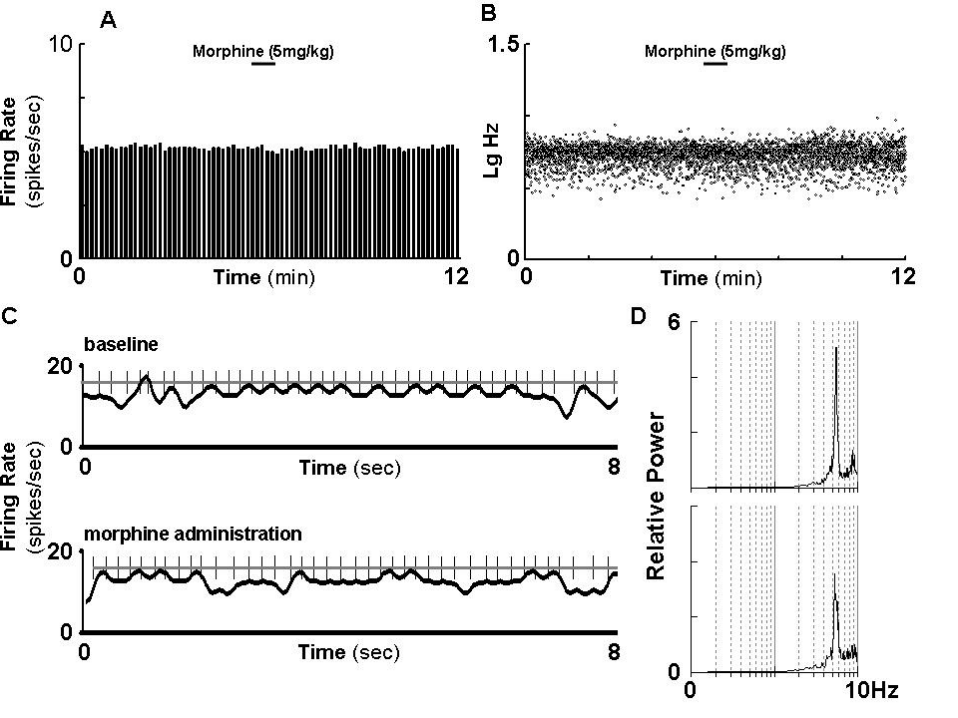
**VTA DA neurons fail to response to subsequent challenge following a single morphine exposure**. Rats received morphine (20 mg/kg, s.c.) or saline, and then challenged with morphine (5 mg/kg, i.v.) at 24 hours. A: firing rate. B: firing rate distribution in same neuron. C: representative segments of firing spike train before (upper panel) and after (low panel) the challenge. D: spectral charts before (upper panel) and after (low panel) the challenge.

**Figure 5:**
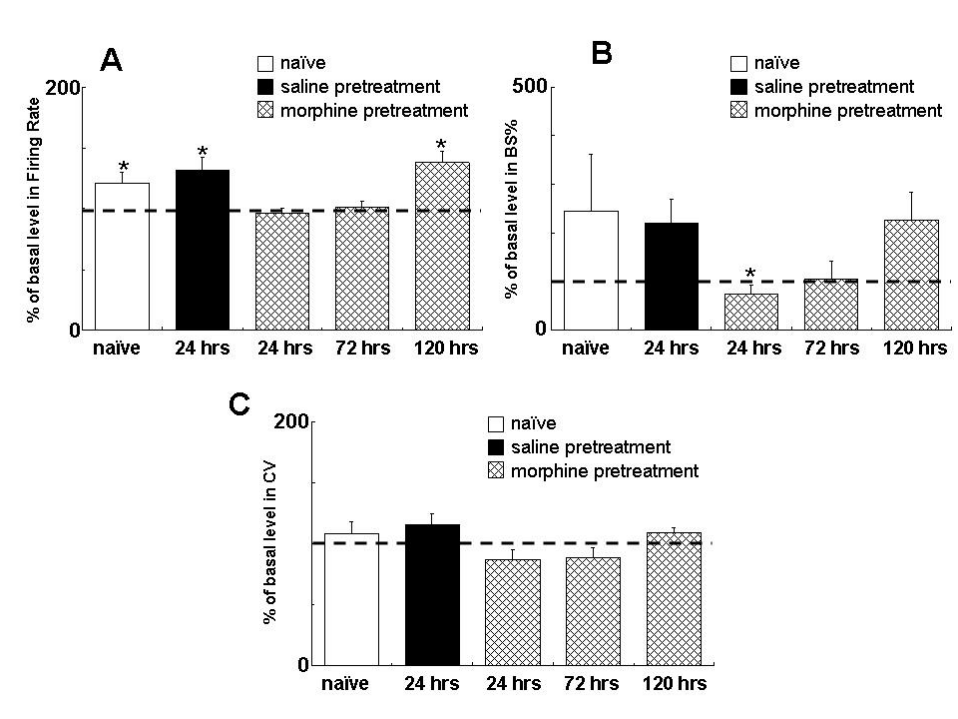
**The firing rate of VTA DA is time-dependently changed in response to subsequent challenge after a single morphine exposure**. Rats received morphine (20 mg/kg, s.c.) or saline, and then challenged with morphine (5 mg/kg, i.v.) 24, 72 or 120 hours later. Data are summary based on at least 5 subjects at each time point in each group and are expressed as % change induced by the challenge. A: firing rate. B: bursting level. C: CV. *p < 0.05, *t*-test.

We next tested how VTA DA neuron responses to subsequent morphine challenge at different time points after initial exposure. In contrast to the activation of VTA DA neurons in response to morphine challenge in naïve rats (Figure. 1), morphine challenge (5 mg/kg, i.v.) at 24 hours after receiving the initial morphine exposure failed to alter the firing rate (-3.5 ± 3.3%, *t*-test, p = 0.322) or CV of ISIs (-13.0 ± 7.9%, *t*-test, p = 0.141) of the VTA DA neurons (Figure. 4 &5). Interestingly, the bursting (-36.4 ± 16.0%, *t *test, p < 0.05) was significantly decreased.

**Figure 6:**
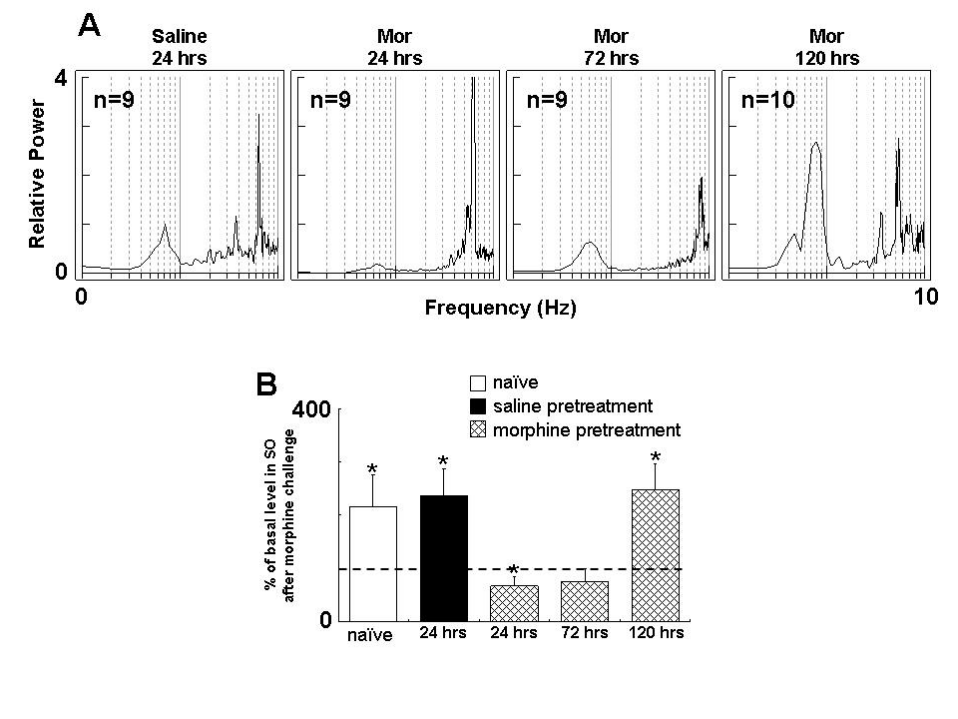
**Slow oscillation (SO) of VTA DA neuron is time-dependently changed in response to morphine challenge after a single morphine exposure**. Rats received morphine (20 mg/kg, s.c.) or saline, and then challenged with morphine (5 mg/kg, i.v.) 24, 72 or 120 hours later. A: representative spectral chart. B: group summary. *P < 0.05, Paired *t *test, compared to respective control.

The failure of the VTA DA neurons in response to morphine challenge lasted at least 72 hours as depicted in firing rate (+0.9 ± 5.1%, *t *-test, p = 0.867), bursting (+6.0 ± 36.4%, *t *test, p = 0.875), CV of ISIs (-11.6 ± 8.5%, *t *-test, p = 0.209) and SO power. This indicated that VTA DA neurons developed a tolerance followed the single dose of morphine pretreatment. However, the response of DA neurons to morphine challenge was restored at 120 hours (Figure. 5): the firing rate was increased by 34.1 ± 10.6% (*t*-test, p < 0.05); The SO power, as shown in Fig. 6, was significantly decreased (-41.8 ± 13.2%, *t*-test, p < 0.05) at 24 h after initial morphine exposure, and was started to recover at 72 h (+24.6 ± 23.7%, *t- *test, p = 0.331), restored at 120 h(118.5 ± 62.0%, *t*-test, p < 0.05). Taken together, the present data demonstrated that single morphine administration results in prolonged unresponsiveness of VTA DA neurons to subsequent morphine challenge which lasted at least three days.

### Inhibition on μ-Opioid-stimulated brain [^35^S]GTPγS binding followed single morphine administration in rats

**Figure 7:**
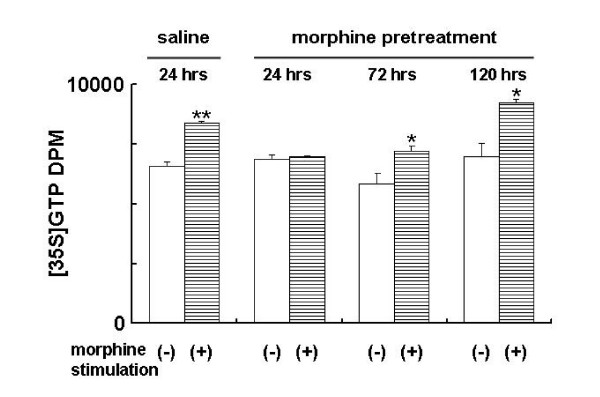
**Morphine-stimulated GTPγS binding is changed after a single morphine exposure**. Rats received morphine (20 mg/kg, s.c.) or saline, and sacrificed 24, 72, or 120 hours later for morphine-stimulated GTPγS binding assay(10 μM of morphine or vehicle in incubation buffer). Data are expressed as mean ± SEM obtained from at least 3 rats. ANOVA, *p < 0.05, **P < 0.01, compared to respective control. (-): no morphine; (+): morphine.

Since desensitization of opioid receptor is the important mechanism for morphine tolerance, we next studied whether the tolerance/dependence developed by single dose of morphine treatment is also associated with the change in opiate receptor-stimulated G protein coupling. We employed [^35^S]GTPγS binding assay to monitor the opioid receptor activation. As shown in Figure 7, the time-course of morphine-stimulated brain [^35^S]GTPγS binding in rats received saline or single dose of morphine was examined at 24, 72, 120 hours after drug or saline administration, respectively. We found that the morphine-stimulated [^35^S]GTPγS binding was increased to 127.4 ± 1.6% over the non-stimulated control (n = 3, p < 0.001) in membrane preparation obtained from saline-pretreated rats. In contrast, morphine failed to stimulate [^35^S]GTPγS binding in brain membrane preparation from rats at 24 hours after initial morphine treatment (101.3 ± 4.8%, n = 3). However, this stimulation was restored at 72 (128.3 ± 3.8%, p < 0.05) and 120 hours (131.8 ± 2.2%, n = 3; p < 0.05), respectively (Figure. 7). This indicated that opioid receptor failed to response to morphine stimulation for a prolonged period of time followed the single dose of morphine administration. This result suggested that development of uncoupling between the opioid receptor and related G-protein may contribute to the tolerance and dependence induced by single morphine administration.

## Discussion

Morphine alters DA neurotransmission that is associated with rewarding circuit. Microdialysis assay has demonstrated an increased DA release in response to acute morphine administration which is generally believed to associate with the positive reinforcing effect of drug[23,26-29]. The underlying pathways for morphine-modulated DA neuron function is suggested to associate with the suppression of GABA inhibitory input to DA neurons in the VTA[30] and an increase in glutamate-mediated excitatory inputs[31]. The direct association between DA neuron firing activities and DA release has been well documented. In agreement with previous observation [15-17], The traditional parameters of the VTA DA neuron activities such as firing rate and bursting were significantly increased in response to acute morphine in naïve rats. In addition, by employing a spectra analysis and cell-per-track technique, we found that the firing pattern and firing population of DA neurons were also changed by acute morphine administration. The present study also provided the first evidence that morphine increases SO of the VTA DA neurons. SO, a recently identified major pattern of DA neuron activity [18], was significantly enhanced upon the acute morphine administration. It is believed that the firing pattern is more important than the firing rate in the regulation of DA release, and oscillatory firing results in oscillatory release of DA from DA neurons[18]. It is thus conceivable that the alteration of SO in VTA DA neuron by acute morphine administration may play an important role in the development of abnormal DA neurotransmission associated with prolonged single morphine-mediated change.

Repeated morphine treatment results in a diminished response of the VTA DA neurons to subsequent morphine challenge over extended period of time [24], which is believed to associate with the development of repeated morphine-induced dependence and tolerance. Likewise, rapid tolerance and dependence induced by a single morphine exposure have also been noticed both in clinical observation and experimental studies[11,14,32]. The underlying mechanisms, however, remain largely unknown. The striking change in the present study is the demonstration of prolonged failure in response to morphine challenge for the VTA DA neuron activities followed initial single morphine exposure which at least sustained for 3 days. The electrophysiological parameters recovered until day 5 followed the single morphine treatment. This failure of VTA DA neurons in response to subsequent morphine challenge after single morphine injection may provide an underlying mechanism for the development of tolerance and dependence followed single morphine treatment at cellular level. However, it is also interested to note that it was reported that in escalation of cocaine self-administration is independent of dopamine levels of nucleus accumbens[33], this indicates that there are mechanistic differences in the development of dependence/tolerance and escalation between morphine *vs *cocaine [34].

In addition to the prolonged changes in VTA DA neuron activity, at receptor levels, we detected an impairment of opiate receptor-G-protein coupling in rat brains with single morphine administration, indicating a desensitization of opioid receptor was also developed in response to single morphine injection (Fig. 7). Desensitization of the opioid receptors followed the repeated morphine or opioid receptor agonst has been repeatedly demonstrated and is linked to the development of tolerance and dependence [24,35-38]. Likewise, the present data provided the first data that opioid receptor also develops a prolonged desensitization with single morphine exposure. It is well known that phosphorylation of opioid receptors is critical for receptor desensitization, the desensitized receptor then internalized via endocytosis, where the receptor either recycles back to membrane or degradation. Altered trafficking of opioid receptor is believed to play an essential role in repeated morphine-elicited tolerance and dependence [37,38]. It appears that a prolonged desensitization of the opioid receptors may underlie the single dose morphine-induced tolerance. It would be interested to study the detail mechanism for how the desensitization alters the receptor recycling which, in turn, contributes to the development of dependence and tolerance followed single dose of morphine.

In summary, the present study demonstrated a prolonged failure in both VTA DA neuron activity and opioid receptors to subsequent morphine challenge followed a single morphine treatment. The further work should focus on the detail molecular events that underlie this change.

## Conclusion

Prolonged VTA DA neuron activation and opiate receptors desensitization followed single morphine exposure may underlie the development of dependence and tolerance which may associate with the alteration of analgesic response and acute addiction of morphine.

## Competing interests

The authors declare that they have no competing interests.

## Authors' contributions

DZ performed the electrophysiological experiments, HZ conducted biochemical assays, GZJ participated experimental design, KHZ and XCZ instructed the project and wrote the manuscript. All authors read and approved the manuscript.
